# ZrO_2_-Assisted QuEChERS-UHPLC-MS/MS for Simultaneous Determination of Four Aflatoxins in Cereals and Soybean Matrices

**DOI:** 10.3390/toxins18040172

**Published:** 2026-04-03

**Authors:** Shusen Liu, Xiaojuan Zheng, Shuo Zhang, Ning Guo, Haijian Zhang, Jie Shi

**Affiliations:** Plant Protection Institute, Hebei Academy of Agriculture and Forestry Sciences/National Collection of Plant-associated Microbes (Hebei)/IPM Innovation Center of Hebei Province/International Science and Technology Joint Research Center on IPM of Hebei Province, Baoding 071000, China; shusenliu@163.com (S.L.); xiaojuanzheng2018@163.com (X.Z.); zhangshuo0121@163.com (S.Z.); guoning433@163.com (N.G.); zhanghaijian322@163.com (H.Z.)

**Keywords:** aflatoxins, zirconium dioxide, QuEChERS, UHPLC-MS/MS, cereal and soybean matrices, food safety

## Abstract

Highly sensitive methods for trace-level aflatoxin determination are indispensable for cereal food safety and public health protection. This study developed a ZrO_2_-assisted QuEChERS-UHPLC-MS/MS method for the simultaneous determination of AFB_1_, AFB_2_, AFG_1_, and AFG_2_ in maize, wheat, rice, and soybean. Systematic optimization identified acetonitrile as the optimal extraction solvent and 10 mg ZrO_2_ in combination with PSA, C_18_, and GCB as the optimal cleanup formulation, providing recoveries of 107.33–111.60%. Chromatographic baseline separation was achieved within 8.0 min using a moderate gradient program. The method exhibited excellent linearity (R^2^ > 0.999) with LODs of 0.15–0.25 µg/kg and LOQs of 0.50–0.75 µg/kg. Negligible matrix effects (0.85–1.02) validated the efficacy of ZrO_2_-assisted cleanup in eliminating co-extractive interferences in maize. Satisfactory accuracy (recoveries of 86.66–111.04%) and precision (RSDs < 14%) were obtained across all matrices. The method demonstrated consistent performance across diverse cereal and soybean matrices, fulfilling international regulatory requirements for routine aflatoxin monitoring in agricultural commodities.

## 1. Introduction

Maize (*Zea mays*), one of the world’s most important staple crops, with annual production exceeding 1.1 billion metric tons in 2020 [1], plays a pivotal role in global food security by supplying over 20% of human caloric intake in many regions, particularly Sub-Saharan Africa, Latin America, and parts of Asia [2]. Consequently, its yield and quality directly impact human food safety and agricultural sustainability. However, maize production faces significant challenges from mycotoxin contamination—hazardous secondary metabolites produced by toxigenic fungi [3]. Of the hundreds of mycotoxins characterized to date [4,5,6], aflatoxins are particularly notorious, primarily produced by toxigenic *Aspergillus* species [7], and designated by the IARC as Group 1 carcinogens due to their potent mutagenic and carcinogenic effects [8,9]. Among over 20 identified aflatoxins, AFB_1_, AFB_2_, AFG_1_, and AFG_2_ are the most prevalent contaminants in food and feed [10], with AFB_1_ and AFB_2_ further metabolized to AFM_1_ and AFM_2_ in dairy cattle, leading to secondary exposure through milk and animal-derived products [3,11].

Aflatoxins may resist severe processes like roasting, extrusion, baking, cooking, and other tough conditions. Due to their stability, it is very difficult to remove them completely from the human diet and animal feed [12,13]. High intake of aflatoxins can cause acute poisoning, while long-term exposure to low levels leads to chronic aflatoxicosis, resulting in liver damage, immunosuppression, and increased risk of mortality in humans and animals [14,15]. Therefore, many countries, regions, and international organizations have implemented strict regulations for aflatoxins in food and feed to maintain the health of individuals [16,17]. For human consumption of maize and maize-based products, the regulated limit for total aflatoxins (AFs), which include AFB_1_, AFB_2_, AFG_1_ and AFG_2_, ranges from 4 to 40 µg/kg. The maximum limit specifically for AFB_1_ varies between 2 and 20 μg/kg [16,17]. The World Health Organization (WHO) has set limits for AFs and AFB_1_ at 10 μg/kg and 5 μg/kg, respectively. In the United States, the maximum allowable limit for AFs in maize products is regulated at 20 μg/kg. China has stipulated that the level of AFB_1_ in edible maize and its derived products must not exceed 20 μg/kg, while the maximum permissible level in maize-based animal feed is set at 50 μg/kg. The European Union (EU) enforces the strictest regulations, with allowable levels for AFs and AFB_1_ capped at 4 μg/kg and 2 μg/kg, respectively. Therefore, it is necessary to establish a reliable and highly sensitive method for detecting trace aflatoxins in maize.

QuEChERS combined with liquid chromatography–tandem mass spectrometry (LC-MS/MS) has become a gold-standard approach for quantifying trace-level analytes in complex matrices. Renowned for its simplified workflow, cost-efficiency, superior selectivity, and exceptional sensitivity, this method enables the simultaneous detection of multiple mycotoxins within a specific matrix. LC-MS/MS has been widely employed for aflatoxin analysis in maize and derived products. Beltrán et al. (2009) developed a UHPLC/MS/MS method for 11 mycotoxins, achieving LODs of 0.1–0.7 μg/kg and LOQs of 0.3–2.5 μg/kg for the four major aflatoxins [18]. Ouakhssase et al. (2019) subsequently reported comparable sensitivity, with LODs of 0.11–0.36 μg/kg and LOQs of 0.36–1.19 μg/kg using optimized LC-MS/MS [19]. More recently, Mbisana et al. (2023) extended this approach to multi-mycotoxin analysis in maize and sorghum by QuEChERS-LC-MS/MS, obtaining LODs of 0.16–0.57 μg/kg and LOQs of 0.53–1.90 μg/kg for aflatoxins [20]. These studies collectively demonstrate the exceptional sensitivity of LC-MS/MS-based methods for aflatoxin detection.

Reliable quantification, however, depends critically on adequate matrix cleanup, which remains challenging for complex matrices with high lipid or pigment content. Conventional QuEChERS sorbents (PSA, C_18_, GCB) often provide insufficient cleanup for such matrices, as evidenced by strong signal enhancement or suppression in maize aflatoxin analysis [21,22]. Zirconium dioxide (ZrO_2_) addresses this limitation through its amphoteric characteristics and abundant Lewis acid sites, enabling selective removal of fatty acids, glycerides, and pigments [23,24]. Previously, ZrO_2_-based sorbents have been applied for pesticide analysis in bovine milk, avocado, and almond, as well as mycotoxin analysis in nuts and baby food [25,26,27,28,29]. However, the efficacy of ZrO_2_ for aflatoxin detection in high-starch, high-protein cereals and oil-rich raw soybean remains unknown. Moreover, the performance of ZrO_2_ combined with conventional sorbents for aflatoxin analysis has not been systematically evaluated.

This study addresses these gaps by optimizing and validating a ZrO_2_-assisted QuEChERS method coupled with UHPLC-MS/MS for the simultaneous determination of aflatoxins in maize, wheat, rice, and raw soybean, representing the first application of ZrO_2_-based cleanup for aflatoxin analysis in these matrices. Specifically, we aimed to (1) systematically optimize ZrO_2_ dosage in combination with conventional sorbents, and (2) establish a robust analytical method with optimized extraction, cleanup, and separation parameters for the rapid, sensitive determination of aflatoxins in cereal and legume matrices.

## 2. Results

### 2.1. Optimization of MS/MS Parameters

**Figure 1:**
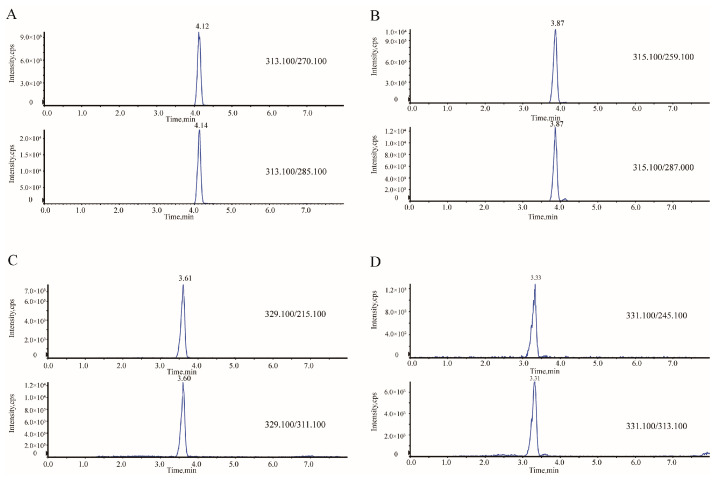
Representative chromatograms of aflatoxins ((**A**) AFB_1_; (**B**) AFB_2_; (**C**) AFG_1_; (**D**) AFG_2_).

Mass spectrometric parameters were optimized by full-scan analysis of a 10 μg/L mixed aflatoxin standard in positive ionization mode, which provided superior signal intensity and abundant [M + H]^+^ precursor ions for all four aflatoxins. For each aflatoxin, the two most abundant product ions were selected based on signal intensity and spectral quality, with the most intense fragment used for quantitation and the second for confirmation. The optimized MS/MS parameters, including precursor/product ions and collision energies, are summarized in Table 1, and representative chromatograms are shown in Figure 1.

### 2.2. Optimization of Sample Preparation and Chromatographic Conditions

#### 2.2.1. Solvent and Sorbent Optimization

**Figure 2:**
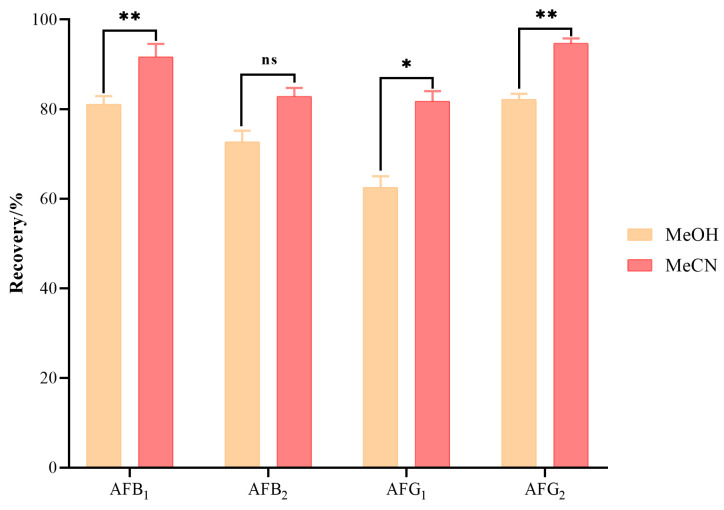
Extraction efficacy of MeOH and MeCN for aflatoxins (*n* = 3). Note: Data are presented as mean ± standard deviation (SD). “ns” indicates no significant. Asterisks indicate significant differences between solvents: * *p* < 0.05, ** *p* < 0.01, paired *t*-test.

The extraction efficiencies of methanol (MeOH) and acetonitrile (MeCN) for AFB_1_, AFB_2_, AFG_1_, and AFG_2_ were evaluated using a blank maize matrix spiked at 0.75 μg/kg. As shown in Figure 2, MeCN exhibited higher extraction efficiency, yielding recoveries of 81.8–94.7% versus 62.5–82.2% for MeOH. Statistical analysis revealed that MeCN provided significantly higher recoveries for AFB_1_, AFG_1_, and AFG_2_. Accordingly, MeCN was selected as the extraction solvent for all subsequent analyses.

**Figure 3:**
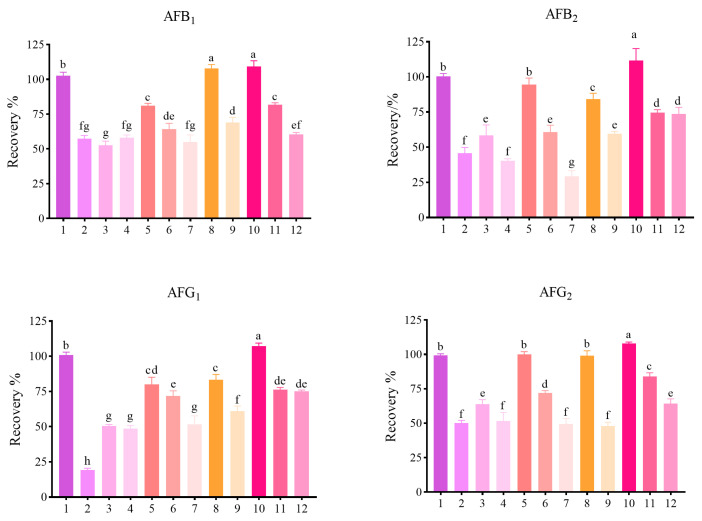
Purification efficacy of sorbent combinations for aflatoxins (*n* = 3). Note: The x-axis values (1–12) denote distinct adsorbent combinations. Data are presented as mean ± standard deviation (SD). Different lowercase letters above the bars indicate statistically significant differences at the 5% level (*p* < 0.05).

The cleanup efficiency of sorbent combinations was systematically evaluated, with particular emphasis on ZrO_2_ dosage optimization. Twelve combinations were tested using blank maize matrix spiked at 2.0 μg/kg. Stepwise optimization revealed distinct dosage-dependent effects (Figure 3). For PSA-only systems (Combinations 1–4), 25 mg provided optimal recoveries of 99.33–102.70% for all four aflatoxins, whereas increasing PSA to 30–50 mg resulted in significant, dose-dependent reductions (*p* < 0.05). With PSA fixed at 25 mg, C_18_ supplementation (Combinations 5–7) demonstrated that 25 mg maintained acceptable recoveries (80.00–100.00%), while higher C_18_ loadings (30–40 mg) caused significant, dose-dependent reductions (*p* < 0.05). The addition of GCB to Combination 5 showed that 10 mg enhanced AFB_1_ recovery to 108.01% while maintaining other aflatoxins above 80.00% (Combination 8); in contrast, 20 mg induced significant adsorption losses (*p* < 0.05), depressing recoveries below 70.00% (Combination 9). Critical optimization was achieved by incorporating ZrO_2_ into Combination 8. The addition of 10 mg ZrO_2_ markedly improved recoveries of all four aflatoxins to 109.33%, 111.60%, 107.33%, and 108.00%, respectively (Combination 10), achieving the highest recoveries among all tested combinations (*p* < 0.05); however, 15–20 mg led to significantly decreased recoveries compared to 10 mg (*p* < 0.05) (Combinations 11–12). Based on comprehensive performance evaluation, the optimal ZrO_2_-assisted formulation (25 mg PSA + 25 mg C_18_ + 10 mg GCB + 10 mg ZrO_2_) was selected for sample purification.

#### 2.2.2. Gradient Elution Optimization

**Figure 4:**
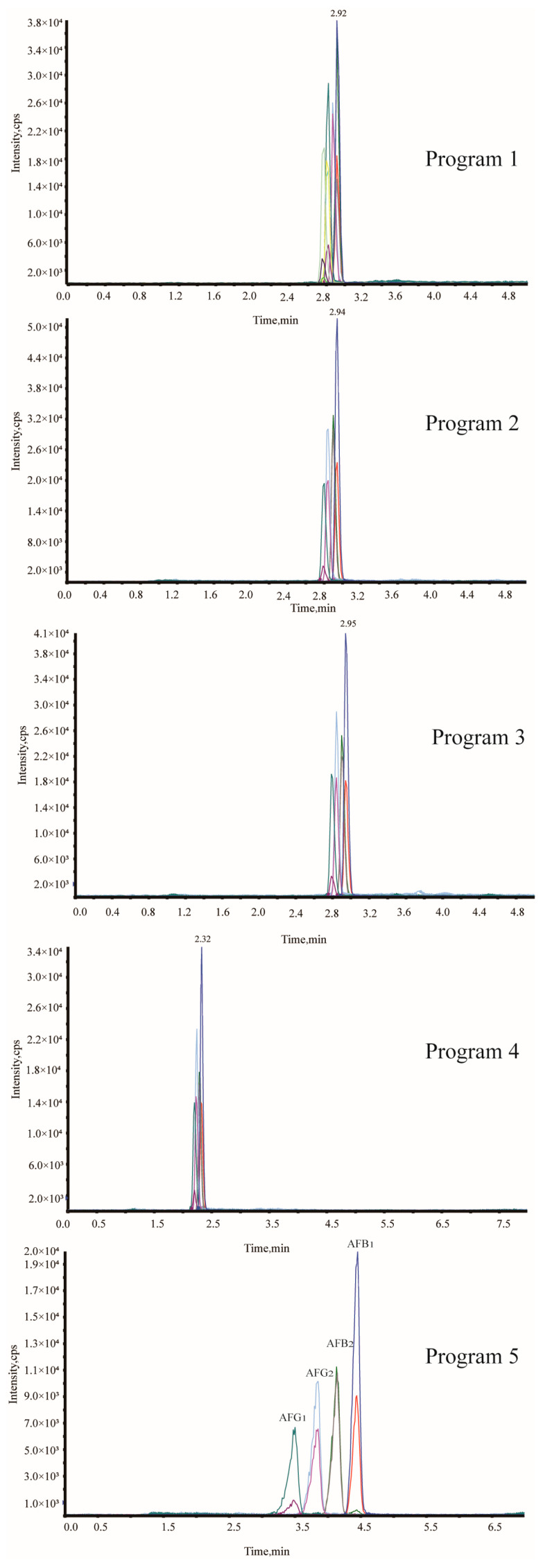
Chromatograms of aflatoxins under optimized gradient elution programs.

To achieve optimal resolution of AFB_1_, AFB_2_, AFG_1_, and AFG_2_, gradient elution conditions were systematically optimized by varying mobile phase composition and program duration. Five candidate programs were evaluated (Table 2), with chromatographic performance shown in Figure 4. Procedures 1–3, employing abbreviated 5.0 min gradients, proved insufficient for complete separation of the four aflatoxins. Although Procedure 4 extended runtime to 8.0 min and modified the initial mobile phase composition from 70:30 to 60:40 (*v*/*v*), thereby reducing retention times, baseline resolution remained unachieved. Baseline separation was ultimately attained with Procedure 5, which implemented a more moderate gradient profile: 0.0–0.5 min, 40% B; 0.5–4.0 min, 70% B; 4.0–5.0 min, 98% B; 5.0–6.0 min, 98% B; 6.0–6.5 min, 40% B; and 6.5–8.0 min, 40% B. This optimized protocol, using a 3.0 μL injection volume, enabled baseline resolution of all four aflatoxins within an 8.0 min analytical cycle.

### 2.3. Performance Validation

#### 2.3.1. Matrix Effect

Matrix effects (MEs) were evaluated by comparing the slopes of matrix-matched and solvent-based calibration curves, with values between 0.8 and 1.2 considered negligible [30,31]. Values outside this range indicate signal suppression or enhancement. In the present study, ME values for all four aflatoxins in the maize matrix ranged from 0.85 to 1.02 (Table 3), indicating negligible MEs and validating the robustness of the developed method.

#### 2.3.2. Linearity and Sensitivity

The linearity and sensitivity of the developed method are summarized in Table 4. Linearity was assessed using a six-point calibration curve over the concentration range of 0.5–100 μg/L. All target aflatoxins exhibited excellent linearity, with determination coefficients (R^2^) > 0.999. Method sensitivity was evaluated based on limits of detection (LODs) and quantification (LOQs), calculated at signal-to-noise (S/N) ratios of 3 and 10, respectively. The LODs were 0.15 μg/kg for AFB_1_ and AFB_2_, and 0.25 μg/kg for AFG_1_ and AFG_2_. Corresponding LOQs were 0.50 μg/kg for AFB_1_ and AFB_2_, and 0.75 μg/kg for AFG_1_ and AFG_2_. These results demonstrate that the method possesses excellent linearity and high sensitivity.

#### 2.3.3. Accuracy and Precision

The accuracy and precision of the developed method were evaluated using blank maize matrices spiked with aflatoxins at three concentration levels (low, medium, and high). As shown in Table 5, satisfactory recoveries were obtained for all four analytes: AFB_1_ (92.28–102.12%), AFB_2_ (94.98–102.40%), AFG_1_ (94.73–98.06%), and AFG_2_ (96.52–111.04%). The corresponding RSD values were 3.86–5.09%, 3.10–5.52%, 3.15–8.72%, and 1.83–5.19%, respectively. These results demonstrate that the method exhibits acceptable accuracy and good precision for the simultaneous determination of AFB_1_, AFB_2_, AFG_1_, and AFG_2_ in maize.

### 2.4. Applicability to Diverse Matrices

To broaden the practical utility of the developed method, its applicability was extended to three additional matrices: wheat, rice, and soybean. As shown in Table 5, satisfactory recoveries were obtained across all matrices at three spiked levels. For AFB_1_, recoveries ranged from 95.59% to 104.51% with RSDs of 3.33–12.91%. AFB_2_ showed recoveries of 86.66% to 98.51% with RSDs of 2.22–9.62%. AFG_1_ and AFG_2_ achieved recoveries of 93.71% to 104.60% and 91.84% to 99.90%, respectively. Notably, higher RSDs were observed at low spiking levels for AFG_1_ in wheat (13.55% at 0.75 μg/kg) and rice (12.56% at 0.75 μg/kg), as well as for AFB_1_ (12.91% at 0.50 μg/kg) and AFG_1_ (12.85% at 0.75 μg/kg) in soybean, suggesting greater analytical variability at these lower concentration ranges. The method demonstrated consistent performance across cereal and soybean matrices, meeting regulatory requirements for accuracy and precision. MEs were also assessed for these matrices. As shown in Table 3, MEs ranged from 0.98 to 1.05 for wheat, 0.96–1.09 for rice, and 1.01–1.08 for soybean, all within acceptable limits. These results confirm that the method is applicable to major cereal grains and soybean in addition to maize.

### 2.5. Results of Real Sample Analysis

The developed method was successfully applied to the analysis of 135 real samples, comprising 60 maize, 45 wheat, and 30 soybean samples. As shown in Table 6, the method effectively detected aflatoxins across different contamination levels and sample types. In maize, AFB_1_ was detected in nine samples (15.00%), all of which exceeded the LOQ, with concentrations ranging from 0.51 to 1.35 μg/kg. AFG_2_ was detected in eight samples (13.33%), with six samples (10.0%) quantifiable at concentrations of 1.44–1.54 μg/kg. AFG_1_ was detected in two samples (3.33%), of which one sample was quantifiable at 0.79 μg/kg. Notably, AFB_2_ was not detected in any maize sample. In wheat, AFB_1_ was detected in twelve samples (26.67%), though only four samples (8.9%) exceeded the LOQ, with concentrations ranging from 0.55 to 0.65 μg/kg. AFB_2_, AFG_1_, and AFG_2_ were each detected in 4–5 samples (8.9–11.1%), but none reached quantifiable levels. In soybean, although AFB_1_ was detected in seven samples (23.3%) above the LOD, no samples exceeded the LOQ. AFB_2_, AFG_1_, and AFG_2_ were not detected in any soybean sample.

## 3. Discussion

### 3.1. Sample Preparation Efficacy

MeCN and MeOH have been widely employed as extraction solvents for mycotoxin extraction. However, Nakhjavan et al. (2020) pointed out that 100% organic solvents are generally ineffective for simultaneous multi-mycotoxin analysis, particularly for polar analytes [32]. Consequently, aqueous–organic mixtures, often with acid additives, are typically used to balance recoveries across diverse toxins in multi-mycotoxin systems. Mbisana et al. (2023) reported that 80% MeCN achieved superior extraction efficiency compared with 80% MeOH and the MeOH/MeCN mixture (50:50, *v*/*v*) for AFB_1_, AFB_2_, AFG_1_, and AFG_2_, albeit with moderate recoveries of only 50–70%. Although supplementation with 0.1–0.5% formic acid marginally improved recoveries, the enhancement remained limited [20]. By contrast, the present study demonstrates that 100% MeCN substantially outperforms these aqueous formulations, achieving recoveries of 81.8–94.7% for the four aflatoxins. These results indicate that 100% MeCN represents the optimal solvent choice for aflatoxin-specific extraction.

The ZrO_2_-assisted QuEChERS procedure developed in this study achieved superior cleanup performance compared to conventional sorbent combinations. The optimal formulation (25 mg PSA + 25 mg C_18_ + 10 mg GCB + 10 mg ZrO_2_) provided excellent recoveries of 109.3%, 111.6%, 107.3%, and 108.0% for AFB_1_, AFB_2_, AFG_1_, and AFG_2_, respectively. While PSA, C_18_, and GCB are conventional QuEChERS sorbents that remove polar interferents (organic acids, pigments, sugars), non-polar lipids, and planar molecules (including aflatoxins), respectively [33,34,35,36], ZrO_2_ provides unique selectivity through its amphoteric characteristics and abundant Lewis acid sites, enabling effective removal of fatty acids, glycerides, and pigments without retaining target aflatoxins [23,24]. The 10 mg ZrO_2_ loading represents a key advancement, as previous studies have not established optimal protocols for this sorbent in aflatoxin analysis. Clear dose-dependent effects were observed for all sorbents in the present study. Excessive PSA (≥30 mg) caused severe analyte loss, with recoveries plummeting to 19.4–63.6%. Similarly, elevated C_18_ and GCB loadings progressively reduced recoveries. For ZrO_2_, 10 mg improved recoveries of AFB_2_, AFG_1_ and AFG_2_ to 107.3–111.6%, whereas increasing the dosage to 15–20 mg significantly suppressed recoveries to 60.5–84.1%. This indicates that while moderate loading effectively removes matrix interferences, excess amounts begin to trap aflatoxins through non-specific interactions. This precise balance maximizes cleanup efficiency while minimizing analyte loss, providing a robust purification strategy for complex matrices.

### 3.2. Optimization of Chromatographic Separation Conditions

The chromatographic separation of all four aflatoxins was systematically optimized to balance resolution, analysis time, and method robustness for routine application. Procedures 1–3 employed abbreviated 5 min gradients with steep elution profiles (70:30 to 2:98 in 2.0 min), which failed to achieve baseline resolution for the four aflatoxins. This outcome resulted from insufficient chromatographic separation of these structurally analogous compounds. Procedure 4 modified the initial mobile phase composition to 60:40 (*v*/*v*) and extended the gradient duration to 5.0 min, thereby reducing analyte retention times. However, incomplete resolution persisted, likely due to inadequate column equilibration between gradient segments. Procedure 5 utilized a moderate gradient profile (60:40 to 2:98 over 5.0 min with a 0.5 min hold at initial conditions) and achieved satisfactory baseline separation. These results demonstrate that controlled elution kinetics, combined with sufficient column equilibration, are essential for resolving planar mycotoxins with similar physicochemical properties. The total cycle time of 8.0 min represents an optimal balance between chromatographic resolution and analytical throughput, fulfilling the requirements for routine regulatory monitoring.

### 3.3. Method Performance and Reliability

The validated method demonstrated satisfactory performance across all evaluated parameters. A linear dynamic range of 0.5–100 μg/L was established for all four aflatoxins, with correlation coefficients (R^2^) ≥ 0.999, ensuring reliable quantification via external standard calibration. Sensitivity, as reflected by LOQs of 0.50–0.75 μg/kg, compares favorably with previously reported methods. Specifically, these LOQs are markedly lower than those reported by Malachová et al. (2014) and Mbisana et al. (2023) (1.90–12.00 μg/kg and 1.19–1.90 μg/kg, respectively) [20,37], while remaining competitive with the optimized methods of Beltrán et al. (2009) and Ouakhssase et al. (2019) (0.30–2.50 μg/kg and 0.36–1.19 μg/kg, respectively) [18,19]. The corresponding LODs (0.15–0.25 μg/kg) further demonstrate adequate detection capability at trace levels. A particularly advantageous feature is the consistent sensitivity across all four aflatoxins, with equivalent LOQs observed for AFB_1_/AFB_2_ and AFG_1_/AFG_2_, respectively. This uniformity, in contrast to the variable performance noted in previous studies [18,19,20,37], underscores the reliability of the established protocol. Furthermore, all LOQs fall well below international regulatory limits for aflatoxins in cereals, confirming the method’s suitability for routine monitoring.

Matrix effects (MEs), arising from co-eluting substances that interfere with the analyte of interest, are essential for evaluating matrix influence on ion intensity [38,39]. Pronounced MEs can impair the linearity, sensitivity, accuracy, and precision of the analytical method [40,41]. In the present study, ME values ranging from 0.85 to 1.02 indicate negligible ion suppression or enhancement, comfortably within the acceptable threshold of 0.8–1.2. This validates the efficacy of the ZrO_2_-assisted QuEChERS cleanup procedure in eliminating co-extractive interferences, underscoring the method’s robustness in maize matrix.

Excellent recoveries and RSDs demonstrated satisfactory accuracy and precision across all four aflatoxins in the maize matrix. Recovery experiments conducted at three spiking levels (0.50, 0.75, and 15.00 μg/kg for AFB_1_ and AFB_2_; 0.75, 2.50, and 15.0 μg/kg for AFG_1_ and AFG_2_) yielded mean recoveries of 92.28–111.04%, fulfilling the EU 2023/2782 acceptance criteria of 70–120% recovery for mycotoxin analysis [42,43]. RSDs were consistently below 10.00% (3.10–5.19%), substantially exceeding the EU legislative precision criteria (RSD < 25.00%) for trace-level mycotoxin analysis [44], thus demonstrating satisfactory reproducibility. These results confirm that the developed method possesses adequate accuracy and precision for reliable quantitative determination of all four aflatoxins in maize.

### 3.4. Application to Diverse Matrices

The successful application across wheat, rice, and soybean highlights the method’s suitability for aflatoxin monitoring in diverse staple crops. Consistently high recoveries (86.66–104.60%) and acceptable precision (RSD < 14% for most analytes) demonstrate the method’s capability to handle matrix variations from high-starch cereals to lipid-rich soybean. The negligible MEs (0.96–1.09, Table 3) observed further support the method’s adaptability across these matrices. Notably, satisfactory performance in soybean, despite its substantially higher lipid and protein content compared to cereals, confirms the robustness of the cleanup strategy against matrix complexity. The elevated variability at low spiking levels, particularly for AFG_1_ in wheat and rice (RSDs of 12.56–13.55%), likely reflects inherent challenges of quantifying near-limit concentrations. Similarly, soybean exhibited higher RSDs at low levels attributable to its complex matrix composition. Nevertheless, all recoveries remained within acceptable regulatory limits, confirming the method’s reliability across the tested matrices.

### 3.5. Practical Applicability of the Method

The application to 135 real samples validates the method’s practical utility for routine aflatoxin monitoring, with particular efficacy demonstrated for maize. The higher proportion of maize samples exceeding the LOQ indicates an elevated contamination risk at the field stage, whereas low detection rates in wheat and soybean demonstrate the method’s adaptability to diverse matrices and effective quality control. Importantly, the quantified levels of AFB_1_, AFG_1_ and AFG_2_ in maize (0.51–1.54 μg/kg) fall substantially below both China’s regulatory limits and the more stringent EU standard, confirming the safety of this primary agricultural product. These results demonstrate the method’s reliability for real sample analysis and highlight its value for aflatoxin monitoring and food safety management.

## 4. Conclusions

A reliable ZrO_2_-assisted UHPLC-MS/MS method was successfully developed and validated for the simultaneous quantification of aflatoxins B_1_, B_2_, G_1_, and G_2_ in maize, wheat, rice, and soybean. Systematic optimization of ZrO_2_ dosage (10 mg) in combination with conventional sorbents (PSA, C_18_, GCB) provided superior cleanup efficiency, evidenced by negligible matrix effects and excellent recoveries. The 8.0 min chromatographic run achieved baseline resolution with satisfactory sensitivity (LOQs 0.50–0.75 µg/kg). The method fulfils international regulatory criteria for accuracy and precision, demonstrating excellent applicability for routine aflatoxin monitoring in various staple crops.

## 5. Materials and Methods

### 5.1. Chemicals and Reagents

Analytical-grade acetonitrile (MeCN), methanol (MeOH), anhydrous magnesium sulfate (MgSO_4_), and sodium chloride (NaCl) were obtained from Tianjin Kemiou Chemical Reagent Co., Ltd. (Tianjin, China), while HPLC-MS grade MeCN and MeOH were purchased from Merck (Burlington, MA, USA). Primary secondary amine (PSA), octadecyl-bonded silica (C_18_), and graphitized carbon black (GCB) were supplied by Shanghai Xinhu Laboratory Equipment Co., Ltd. (Shanghai, China), and zirconium oxide (ZrO_2_) nanoparticles were supplied by Shanghai Yaoyi Alloy Materials Co., Ltd. (Shanghai, China). Standard solutions of AFB_1_ (100 μg/mL), AFB_2_, AFG_1_, AFG_2_ (10 μg/mL) were purchased from Beijing Manhage Biotechnology Co., Ltd. (Beijing, China).

### 5.2. Preparation of Standard Solutions

A 1 μg/mL standard mixture solution of AFB_1_, AFB_2_, AFG_1_ and AFG_2_ was prepared by diluting individual standard solutions with acetonitrile. All standard solutions were stored in amber glass vials with screw caps at −20 °C and allowed to equilibrate to room temperature (~25 °C) for 15 min prior to use.

### 5.3. Sample Preparation

#### 5.3.1. Sample Collection and Pretreatment

Maize kernels used for method development and validation were provided by the Plant Protection Institute of Hebei Academy of Agriculture and Forestry Sciences (Baoding, China), and real samples were collected directly from the field in Hebei Province. Wheat flour, rice and soybean samples were purchased from local supermarkets in Baoding City, China. All samples, except wheat flour (already powdered), were homogenized by grinding 1000 g aliquots in a laboratory grinder and passing through a 20-mesh sieve.

#### 5.3.2. Optimization of Extraction Solvent

For extraction optimization, approximately 2.00 ± 0.01 g of homogenized sample was weighed into a 50 mL polypropylene centrifuge tube and fortified with 1 mL of mixed aflatoxin standard solution. The mixture was allowed to equilibrate for 15 min at room temperature prior to solvent addition. To evaluate solvent effects, 10 mL of MeOH or MeCN was added, and the mixture was homogenized for 3 min using a vortex oscillator (SN-DMT-2500, SUNNE, Shanghai, China), followed by sonication at 80 kHz for 30 min (SB-800D, Scientz, Ningbo, China). Subsequently, 1 g of NaCl and 1 g of MgSO_4_ were added, the mixture was vortexed for 3 min and centrifuged at 8000 rpm (2-16R, Hengnuo, Changsha, China) for 5 min. The supernatant was collected for further analysis.

#### 5.3.3. Optimization of Cleanup Adsorbents

To optimize cleanup, twelve combinations of dispersive solid-phase extraction (dSPE) adsorbents comprising PSA, C_18_, GCB, and ZrO_2_ were evaluated (Table 7). A 1.5 mL aliquot of the extract was mixed with the dSPE adsorbents, vortexed for 5 min, and centrifuged at 10,000 rpm for 5 min at 4 °C (CF1524R, SCILOGEX, Rocky Hill, CT, USA). Finally, 1 mL of the resulting supernatant was passed through a 0.22 μm PTFE syringe filter, and the filtrate was analyzed by UHPLC-MS/MS.

### 5.4. Chromatographic and Mass Spectrometry Conditions

#### 5.4.1. Chromatographic Conditions

Chromatographic separation was performed on a UHPLC system (SCIEX, Framingham, MA, USA) equipped with an ExionLC AD Pump, an ExionLC AD autosampler, and an ExionLC AD column oven. An ACQUITY UPLC BEH C18 column (50 mm × 2.1 mm, 1.7 μm, Waters, Milford, MA, USA) was used, and the column oven temperature was set at 40 °C. The mobile phase consisted of water (A) and methanol (B). The gradient program was optimized for baseline separation of the four aflatoxins (Table 2). The flow rate was 0.20 mL/min, and the injection volume was 3.0 μL.

#### 5.4.2. Mass Spectrometry Conditions

MS/MS detection was performed using an electrospray ionization (ESI) source (Turbo V™, SCIEX, Singapore) coupled with a triple quadrupole mass spectrometer (AB SCIEX Triple Quad^TM^ 3500, Singapore). The ESI parameters were as follows: ion spray voltage, 5500 V; source temperature, 550 °C; gas 1 (nebulizer), 50 psi; gas 2 (heater), 55 psi; curtain gas, 30 psi. Quantification was conducted in positive ion mode using multiple reaction monitoring (MRM). Data acquisition was performed using Analyst TF 1.7.3 software (SCIEX, Shanghai, China). The MRM parameters are summarized in Table 1.

### 5.5. Method Validation

Linearity, limits of detection (LOD), and quantification (LOQ), accuracy, and precision were evaluated. Calibration curves were constructed using six concentration levels of the standard mixture solution (0.5, 1, 5, 25, 50, and 100 μg/L). LOD and LOQ were determined based on signal-to-noise ratios (S/N) of 3 and 10, respectively. To assess accuracy and precision, blank maize samples (2.00 ± 0.01 g) were spiked with aflatoxin standards at three concentration levels: 0.5, 0.75, and 15 μg/kg for AFB_1_ and AFB_2_; and 0.75, 2.5, and 15 μg/kg for AFG_1_ and AFG_2_. Six replicates were analyzed at each level, and recoveries and relative standard deviations (RSDs) were calculated.

### 5.6. Cross-Matrix Performance Evaluation

To evaluate the versatility of the developed method for aflatoxin analysis across diverse food matrices, finely ground wheat, rice, and soybean flours were spiked at three concentration levels (identical to those used in the method validation) and analyzed using the validated procedure. Recoveries and RSDs were determined for each matrix.

### 5.7. Real Sample Detection

The developed method was applied to real samples of maize, wheat, and soybean. Sixty maize samples were collected prior to harvest from six distinct regions in Hebei Province. Additionally, 45 wheat flour samples and 30 soybean samples were purchased from local supermarkets in Baoding City. All samples were processed and analyzed under the optimized conditions.

### 5.8. Data Analysis

All data were quantitatively analyzed and processed using the SCIEX OS software (version 2.1.6.59781, SCIEX, Shanghai, China). Recovery calculations and matrix effect evaluations were performed using Microsoft Excel 2016 (Microsoft Corporation, Redmond, WA, USA). Statistical analyses were conducted using SPSS 16.0 (IBM, Chicago, IL, USA), with a paired *t*-test for solvent comparison and one-way ANOVA followed by Duncan’s multiple range test for sorbent evaluation (*p* < 0.05). Graphing was performed using GraphPad Prism 9.0 (GraphPad Software, San Diego, CA, USA).

## Figures and Tables

**Table 1:** Optimized MS/MS parameters for aflatoxin analysis.

Aflatoxins	Retention Time (min)	Precursor Ion (*m*/*z*)	Product Ion (*m*/*z*)	Collision Energy (V)	Declustering Potential (V)
AFB_1_	4.14	313.10	285.10	31.00	80.00
	4.12		270.10	29.00	
AFB_2_	3.87	315.10	287.00	38.00	110.00
	3.87		259.10	42.00	
AFG_1_	3.60	329.10	311.10	32.00	115.00
	3.61		215.10	45.00	
AFG_2_	3.31	331.10	313.10	37.00	120.00
	3.33		245.10	54.00	

**Table 2:** The parameters of gradient elution procedures.

Procedure 1		Procedure 2		Procedure 3		Procedure 4		Procedure 5	
Time (min)	A:B (*v*/*v*)	Time (min)	A:B (*v*/*v*)	Time (min)	A:B (*v*/*v*)	Time (min)	A:B (*v*/*v*)	Time (min)	A:B (*v*/*v*)
0.0	70:30	0.0	70:30	0.0	70:30	0.0	60:40	0.0	60:40
0.2	70:30	0.2	70:30	0.2	70:30	0.2	20:80	0.5	60:40
1.0	20:80	1.0	20:80	1.0	20:80	5.0	20:80	4.0	30:70
2.0	2:98	2.0	5:95	2.0	10:90	5.5	2:98	5.0	2:98
3.0	2:98	3.0	2:98	3.0	2:98	6.5	2:98	6.0	2:98
3.5	70:30	3.5	70:30	3.5	70:30	6.6	60:40	6.5	60:40
5.0	70:30	5.0	70:30	5.0	70:30	8.0	60:40	8.0	60:40

**Table 3:** Matrix effects of aflatoxins in maize, wheat, rice, and soybean matrices.

Aflatoxins	Maize	Wheat	Rice	Soybean
AFB_1_	1.02	1.05	1.06	1.08
AFB_2_	0.94	1.00	0.96	1.01
AFG_1_	0.85	0.98	1.09	1.08
AFG_2_	1.00	1.04	1.07	1.02

**Table 4:** Calibration curve, LOD and LOQ of the maize matrix.

Aflatoxins	Regression Equation	R^2^	LOD (μg/kg)	LOQ (μg/kg)
AFB_1_	Y = 15,066.85 X + 689.39	0.999	0.15	0.50
AFB_2_	Y = 9745.42 X + 512.48	0.999	0.15	0.50
AFG_1_	Y = 19,749.77 X + 47.75	0.999	0.25	0.75
AFG_2_	Y = 343.00 X + 100.89	0.999	0.25	0.75

**Table 5:** Accuracy and precision data for aflatoxins in different matrices (*n* = 6).

Aflatoxins	Matrices	Spiked Level/(μg/kg)	Recovery /(%)	RSD /(%)	Spiked Level/(μg/kg)	Recovery /(%)	RSD /(%)	Spiked Level/(μg/kg)	Recovery /(%)	RSD /(%)
AFB_1_	maize	0.50	92.28	5.09	0.75	100.61	3.86	15.00	102.12	4.99
	wheat	0.50	98.06	4.28	0.75	97.38	3.33	15.00	104.51	5.25
	rice	0.50	97.13	6.17	0.75	101.46	3.86	15.00	98.48	6.83
	soybean	0.50	101.80	12.91	0.75	95.59	4.78	15.00	96.68	6.27
AFB_2_	maize	0.50	94.98	3.10	0.75	102.40	5.10	15.00	96.53	5.52
	wheat	0.50	96.09	9.15	0.75	96.48	4.39	15.00	98.51	4.79
	rice	0.50	96.73	6.40	0.75	86.66	2.22	15.00	97.04	3.02
	soybean	0.50	96.55	9.62	0.75	89.37	2.42	15.00	88.07	3.22
AFG_1_	maize	0.75	94.73	3.15	2.50	96.34	8.72	15.00	98.06	3.78
	wheat	0.75	101.56	13.55	2.50	104.60	7.30	15.00	94.24	5.18
	rice	0.75	98.66	12.56	2.50	101.16	7.27	15.00	93.71	5.64
	soybean	0.75	95.80	12.85	2.50	102.30	5.33	15.00	101.05	4.15
AFG_2_	maize	0.75	111.04	5.19	2.50	98.92	4.45	15.00	96.52	1.83
	wheat	0.75	98.41	8.37	2.50	95.56	2.93	15.00	99.90	6.24
	rice	0.75	97.00	3.20	2.50	93.76	5.91	15.00	98.56	5.58
	soybean	0.75	95.10	4.20	2.50	97.76	2.66	15.00	91.84	9.67

**Table 6:** Analysis of aflatoxins in real samples.

Samples	Aflatoxins	No. of Samples ≥ LOD	No. of Samples ≥ LOQ	Concentration Range (μg/kg, Samples ≥ LOQ)
Maize	AFB_1_	9	9	0.51–1.35
	AFB_2_	0	0	/
	AFG_1_	2	1	0.79
	AFG_2_	8	6	1.44–1.54
Wheat	AFB_1_	12	4	0.55–0.65
	AFB_2_	5	0	/
	AFG_1_	4	0	/
	AFG_2_	5	0	/
Soybean	AFB_1_	7	0	/
	AFB_2_	0	0	/
	AFG_1_	0	0	/
	AFG_2_	0	0	/

**Table 7:** Combinations of solid-phase extraction adsorbents.

Combinations	PSA/mg	C_18_/mg	GCB/mg	ZrO_2_/mg
1	25	/	/	/
2	30	/	/	/
3	40	/	/	/
4	50	/	/	/
5	25	25	/	/
6	25	30	/	/
7	25	40	/	/
8	25	25	10	/
9	25	25	20	/
10	25	25	10	10
11	25	25	10	15
12	25	25	10	20

## Data Availability

The original contributions presented in this study are included in the article. Further inquiries can be directed to the corresponding author.

## Abbreviations

The following abbreviations are used in this manuscript:

AFB_1_	Aflatoxin B_1_
AFB_2_	Aflatoxin B_2_
AFG_1_	Aflatoxin G_1_
AFG_2_	Aflatoxin G_2_
MeOH	Methanol
MeCN	Acetonitrile
dSPE	Dispersive solid-phase extraction
PSA	Primary secondary amine
C_18_	Octadecyl-bonded silica
GCB	Graphitized carbon black
ZrO_2_	Zirconium oxide nanoparticles
MgSO_4_	Anhydrous magnesium sulfate
NaCl	Sodium chloride
UHPLC-MS/MS	Ultra-High-Performance Liquid Chromatography–Tandem Mass Spectrometry
ESI	Electrospray ionization
MRM	Multiple reaction monitoring
S/N	Signal-to-noise ratio
LODs	Limits of detection
LOQs	Limits of quantification
RSD	Relative standard deviation
